# Parental Socioeconomic Status, Childhood Asthma and Medication Use – A Population-Based Study

**DOI:** 10.1371/journal.pone.0106579

**Published:** 2014-09-04

**Authors:** Tong Gong, Cecilia Lundholm, Gustaf Rejnö, Carina Mood, Niklas Långström, Catarina Almqvist

**Affiliations:** 1 Department of Medical Epidemiology and Biostatistics, Karolinska Institutet, Stockholm, Sweden; 2 Swedish Institute for Social Research, Stockholm University, Stockholm, Sweden; 3 Astrid Lindgren Children’s Hospital, Lung and Allergy Unit, Karolinska University Hospital, Stockholm, Sweden; Taipei City Hospital, Taiwan

## Abstract

**Background:**

Little is known about how parental socioeconomic status affects offspring asthma risk in the general population, or its relation to healthcare and medication use among diagnosed children.

**Methods:**

This register-based cohort study included 211,520 children born between April 2006 and December 2008 followed until December 2010. Asthma diagnoses were retrieved from the National Patient Register, and dispensed asthma medications from the Prescribed Drug Register. Parental socioeconomic status (income and education) were retrieved from Statistics Sweden. The associations between parental socioeconomic status and outcomes were estimated by Cox proportional hazard regression.

**Results:**

Compared to the highest parental income level, children exposed to all other levels had increased risk of asthma during their first year of life (e.g. hazard ratio, HR 1.19, 95% confidence interval, CI 1.09–1.31 for diagnosis and HR 1.17, 95% CI 1.08–1.26 for medications for the lowest quintile) and the risk was decreased after the first year, especially among children from the lowest parental income quintile (HR 0.84, 95% CI 0.77–0.92 for diagnosis, and HR 0.80, 95% CI 0.74–0.86 for medications). Further, compared to children with college-educated parents, those whose parents had lower education had increased risk of childhood asthma regardless of age. Children with the lowest parental education had increased risk of an inpatient (HR 2.07, 95% CI 1.61–2.65) and outpatient (HR 1.32, 95% CI 1.18–1.47) asthma diagnosis. Among diagnosed children, those from families with lower education used fewer controller medications than those whose parents were college graduates.

**Conclusions:**

Our findings indicate an age-varying association between parental income and childhood asthma and consistent inverse association regardless of age between parental education and asthma incidence, dispensed controller medications and inpatient care which should be further investigated and remedied.

## Introduction

Asthma is the most prevalent chronic disease among children and is associated with morbidity, substantial healthcare resource use and parental absence from work [1], [2]. Following an increase in prevalence over the last decades, recent data suggest that the prevalence has plateaued at 6–9% [3]. Despite the considerable genetic contribution [4], there are likely several additional social and environmental factors involved in the cause and exacerbation of childhood asthma [5], [6]. Several studies have tried to explain how socioeconomic status (SES) influences the development of asthma but with discrepant results. Most studies have reported that children in families with low SES (measured by parental occupation, education or family income), have an increased asthma risk even after adjustment for known confounders such as prenatal maternal smoking, indoor allergens, and maternal stress [7]–[9]. Other studies, however, found no relationship with parental SES [10]–[12]. These diverse results could be due to different study designs, small sample sizes, or varying measures of SES [13].

Current global and national treatment guidelines emphasize use of controller medications such as inhaled corticosteroids (ICS) or leukotriene receptor antagonists (LTRA) for asthma control [14], [15]. However, clinical research has shown that parental SES strongly impacts their offspring’s quality of care and asthma control [16]–[18]. Among studies investigating SES and asthma pharmacotherapy, none have been done in children younger than age 5.

In this study we investigated whether parental income and education were associated with the risk of childhood asthma in a nationwide register-based cohort of preschool children. We also investigated whether parental income and education were associated with an inpatient or outpatient diagnosis and use of controller medications among all diagnosed children identified from the cohort.

## Methods

The Swedish National Board of Health and Welfare holds a number of registers covering health information. The universal use of the Personal Identity Number (PIN), a unique identifier for each resident, enables unambiguous linkage between these registers and those covering socioeconomic information held by Statistics Sweden. We conducted a prospective population-based cohort study among children aged 0–4 years in Sweden, using data from the Medical Birth Register (MBR), the Multi-Generation Register (MGR), the National Patient Register (NPR), the Prescribed Drug Register (PDR), and the longitudinal integration database for health insurance and labour market studies (LISA by Swedish acronym), linked through each individual’s unique PIN. The regional ethical review board in Stockholm, Sweden granted permission for the study and all individual’s information was anonymized and de-identified prior to analysis.

### Study design and participants

From the MBR (reporting more than 99% of all births in Sweden since 1973 [19]), we included all children born between 1 April 2006 and 31 December 2008 (n = 288,872) in the cohort; this ensured full coverage of dispensed medications for children since birth and their mothers’ pregnancy period from the PDR (established on 1 July 2005). Fathers were linked to their children through the MGR. Children of parents who were born abroad and migrated to Sweden after 15 years of age were excluded from the study (n = 77,352, 26.8%) to ensure full information on highest attained level of education in Statistics Sweden. The children included in the study were followed from birth to 31 December 2010.

### Measurements of socioeconomic status

Parental SES (annual disposable income and education) data were obtained from the LISA database at yearly intervals from the child’s birth year. LISA includes all individuals 16 years and older living in Sweden and integrates labour market, educational and social sector data from several nationwide, annually updated registers. Firstly, parental SES was measured by annual disposable income, which includes individual net benefits after deduction of debits such as taxes, repaid study allowance, and paid maintenance support. The household level disposable incomes were summed up and adjusted for consumption weights [20] to calculate the disposable income per consumption unit, make it comparable for different family sizes and compositions, and converted to euros [21]. Annual disposable incomes per consumption unit were finally divided into quintiles. Secondly, parental SES was also measured by highest education attained within each couple and categorized as compulsory school (≤9 years of education), high school (10–12 years), some college (13–14 years), and college graduate or higher education (≥15 years).

### Outcome measures

There are two common care-seeking pathways for asthma conditions, one of which is a primary care physician occasionally followed by referral to a hospital specialist and the other contact with a specialist directly depending on symptoms and availability. Incident asthma was defined either by diagnosis from the NPR, which covers more than 99% of all hospital discharges and about 80% of all outpatient specialist visits, or by filling two prescriptions for asthma medication from the PDR, which contains data on all prescribed medication dispensed in Swedish pharmacies since 1 July 2005. Both asthma measures have been previously validated in population-based analyses [22]. From the NPR, asthma was ascertained if there was at least one hospital admission or outpatient department visit containing a primary diagnosis of asthma (code J45–46 according to the 10^th^ version of the International Classification of Diseases (ICD-10)). The level of healthcare resource used at diagnosis was categorized as inpatient or outpatient care. Time to the next visit at inpatient care after the first diagnosis was measured as time from the first visit at inpatient (discharge date) or outpatient (visit date) care to the date of the next admission to in-patient care with an asthma diagnosis. From the PDR, incidence date of asthma medication was defined as the date of the first dispensed medication followed by at least one more record of any of the four anti-asthma medications within 24 months: ICS, β2 agonists, combination products or LTRA (identified by Anatomical Therapeutic Chemical (ATC) codes R03AC, R03AK, R03BA, and R03DC, respectively). The average daily dose was defined and categorized in each of three groups: ICS, β2-agonists, and LTRA. The combination products (R03AK) were split into both ICS and β2-agonists according to corresponding proportion of active ingredients. The daily dose of each type of medication was calculated as the total amount of active ingredients across all dispensed packages of this medication divided by number of days from the date of first asthma diagnosis to end of follow-up.

### Covariates

Other independent variables included were the child’s age, gender, number of children born in the family during the study period, metropolitan areas (Stockholm, Gothenburg, Malmö vs the rest of Sweden) and healthcare regions based on residential addresses at birth year (Stockholm-Gotland, Uppsala-Örebro, Northern, Western, Southeastern, and Southern Sweden), to account for shared healthcare resources between counties and regional differences of asthma prevalence. Maternal characteristics included were age at first antenatal care visit, marital status (single, married/cohabiting), maternal smoking during pregnancy (0, 1–9 or ≥10 cigarettes per day). Delivery characteristics included were preterm birth (≤37 weeks of gestational age), low birth weight (<2,500 grams) and parity (first child or not first child). Maternal and delivery characteristics were retrieved from the MBR.

### Statistical analyses

Cox proportional hazards regression was used for the following (yes/no) endpoints: asthma diagnosis, at least two dispensed asthma medications (since the first active prescription), an inpatient asthma diagnosis, and an outpatient asthma diagnosis. Observations were censored at the date of death, migration, or end of follow-up (31 Dec 2010). Parental income and education were modelled as time-dependent variables, with yearly updates. Cumulative hazards for childhood-onset asthma and SES indicators were estimated with the Nelson–Aalen method. In order to examine the independent and joint effects of each SES indicator on asthma outcomes, we performed all analyses with adjustment for one single indicator, mutual adjustment (education in models of income and income in models of education) and interaction terms for education and income. Wald test was used to test for interaction between education and income and between SES measures and preterm birth. Attained age was the underlying analysis time scale. The proportional hazards assumption was checked with a test based on the Schoenfeld residuals as well as graphical examination. When the proportional hazards assumption was violated for the exposure variables, time (age) dependent hazard ratios (HR) were estimated. In the sub-cohort of children with at least one asthma diagnosis from NPR or at least 2 dispensed asthma medications regardless of diagnosis, the associations between parental income and education at the time of first diagnosis/medication and medication dosage, were analysed using linear regression with logarithmic transformed daily average dose of medication as outcome variables (among those with the corresponding medication). Children with at least one asthma diagnosis from NPR were further analysed using Cox regression model with parental income and education as time-dependent variables for the risk of a visit at inpatient care after a first diagnosis, with time from first inpatient/outpatient diagnosis as analysis time scale. Directed acyclic graph was used to determine potential confounders for different models [23]. For all regression analyses we presented the estimates adjusted for child’s gender, parity, maternal age and marital status during pregnancy, healthcare regions, and metropolitan areas. We also fitted models with further adjustment for maternal smoking during pregnancy, low birth weight, and preterm birth. Analyses of parental SES and next visit at inpatient care after first diagnosis as well as of parental SES and average doses of controller medications were further adjusted for levels of healthcare use at previous hospital visit. Sensitivity analysis was performed for severe asthma defined as ≥3 medications within 12 months. As some families contribute more than one child, robust standard errors were used to account for family clustering. The measures of associations presented were hazard ratios (HR) for Cox regression models and exp(β) for linear regression models on log transformed data together with 95% confidence intervals (CI). Stata 12.0 was used for all analyses and a 5% significance level.

## Results

Table 1 shows characteristics at birth and follow-up in the full cohort of 211,520 children and among those diagnosed with asthma. The average person-years at risk were 3.2 in the study population. The cumulative incidence of an asthma diagnosis was 6.6% during the study period and that of having at least two dispensed asthma medications was 10.6%. Mean annual disposable income at child birth was 192,436 SEK (20,169 EUR, 2010 annual exchange rate). There were 2.6% of households that had compulsory schooling as their highest education, 38.4% had high school, 13.7% had some college and 45.2% had completed college or a higher level of education. Children diagnosed with asthma were more often male, born preterm or with low birth weight and had more often been exposed to maternal smoking during pregnancy.

**Table 1:** Study population and background characteristics related to childhood asthma (diagnosis and > = 2 medications) in a Swedish cohort of 211,520 children aged 0–4.5 years.

		All childrenborn during2006.04.01–2008.12.31	Children with ≥1asthma diagnosis		Children with ≥2asthma medicationswithin 2 years
N		211,520	13,990		22,520
**Person-years at risk** **till end of follow-up**			683,448		670,107
**Child’s characteristics**		n (%)*			
Gender	Male	109,012 (51.5)	8,978 (64.2)		13,934 (61.9)
	Female	102,508 (48.5)	5,012 (35.8)		8,587 (38.1)
Age (end of follow-up or time at first event)*	Mean± SD	2.9±0.8	3.1±0.8		3.0±0.8
Preterm birth	Yes (≤37 weeks)	12,876 (6.1)	1,485 (10.6)		2,295 (10.2)
	No (>37 weeks)	198,534 (93.9)	12,500 (89.4)		20,211 (89.8)
	Missing	110 (0.1)	5 (0.0)		14 (0.1)
Low birth weight	Yes (<2,500 g)	8,801 (4.2)	977 (7.0)		1,598 (7.1)
	No (≥2,500 g)	202,438 (95.7)	12,989 (92.8)		20,884 (92.7)
	Missing	281 (0.1)	24 (0.2)		38 (0.2)
Parity	First	95,394 (45.1)	5,410 (38.7)		9,465 (42.0)
	Not first	116,126 (54.9)	8,580 (61.3)		13,055 (57.9)
Living inmetropolitan areas at age 1	Yes	80,806 (38.2)	5,690 (40.7)		9,001 (40.0)
	No	129,153 (61.1)	8,282 (59.2)		13,494 (59.9)
	Missing	1,561 (0.7)	18 (0.1)		25 (0.1)
Healthcare regions	Stockholm-Gotland	46,571 (22.0)	3,584 (25.6)		5,563 (24.7)
	Uppsala-Örebro	49,586 (23.4)	2,836 (20.3)		4,465 (19.8)
	Southeast	22,456 (10.6)	1,294 (9.3)		2,408 (10.7)
	South	40,668 (19.2)	3,064 (21.9)		5,100 (22.7)
	West	35,873 (17.0)	2,220 (15.9)		3,424 (15.2)
	North	14,805 (7.0)	974 (7.0)		1,535 (6.8)
	Missing	1,561 (0.7)	18 (0.1)		25 (0.1)
Asthmamedications (≥2 medications)	No	189,000 (89.4)	3,102 (22.2)		-
	Yes	22,520 (10.6)	10,888 (77.8)		-
Any asthmadiagnosis (≥1 diagnosis)	No	197,530 (93.4)	-		11,632 (51.6)
	Yes	13,990 (6.6)	-		10,888 (48.4)
**Family characteristics**					
Annual disposable incomeper consumption unit atchild birth	Lowest (≤13,942 EUR)	42,329 (20.0)	3,116 (22.3)		4,517 (20.1)
	Lower middle (13,942–16,725 EUR)	42,244 (20.0)	3,085 (22.1)		4,787 (21.3)
	Middle (16,725–19,490 EUR)	42,289 (20.0)	2,873 (20.5)		4,568 (20.3)
	Upper middle (19,490–23,750 EUR)	42,268 (20.0)	2,582 (18.5)		4,427 (19.6)
	Highest (>20,093 EUR)	42,269 (20.0)	2,332 (16.7)		4,216 (18.7)
	Missing	121 (0.0)	2 (0.0)		5 (0.0)
Income quintile changesduring follow-up	Yes	135,755 (64.2)	9,281 (66.3)		14,770 (65.6)
	No	75,218 (35,6)	4,682 (33.5)		7,723 (34.3)
	Missing	547 (0.3)	27 (0.2)		27 (0.1)
Highest education betweenparents at child birth	Compulsory school	5,591 (2.6)	532 (3.8)		713 (3.2)
	High school	81,206 (38.4)	6,037 (43.2)		9,377 (41.7)
	Some college (<3 years)	28,903 (13.7)	1,817 (13.0)		2,956 (13.1)
	College graduates or higher	95,630 (45,2)	5,597 (40.0)		9,462 (42.0)
	Missing	190 (0.1)	7 (0.1)		12 (0.1)
Education changesduring follow-up	Yes	3,969 (1.9)	296 (2.1)		430 (1.9)
	No	206,935 (97.8)	13,660 (97.6)		22,056 (97.9)
	Missing	616 (0.3)	34 (0.2)		34 (0.2)
Maternal age at delivery*	Mean± SD	30.4±5.1	30.0±5.0		30.3±5.0
Mother’s smoking during pregnancy	Not smoking	186,093 (88.0)	11,690 (83.6)		19,195 (85.2)
	1–9 cigarettes/day	10,651 (5.0)	1,049 (7.5)		1,456 (6.5)
	≥10 cigarettes/day	3,185 (1.5)	368 (2.6)		487 (2.2)
	Missing	11,591 (5.5)	883 (6.3)		1,382 (6.1)
Mother’s marital status	Single	16,064 (7.6)	1,168 (8.4)		1,794 (8.0)
	Married or cohabiting	194,107 (91.8)	12,766 (91.3)		20.639 (91.7)
	Missing	1,349 (0.6)	56 (0.4)		87 (0.4)
Family history of asthma	Yes (either parent)	32,822 (15.5)	3,619 (25.9)		5,811 (25.8)
	No	178,698 (84.5)	10,371 (74.1)		16,709 (74.2)
Number of childrenborn in thefamily during thestudy period	1	179,665 (84.9)	11,705 (83.7)		19,050 (84.6)
	2	30,983 (14.7)	2.198 (15,7)		3,347 (14.9)
	≥3	872 (0.4)	87 (0.6)		123 (0.5)

*n (%) indicated the frequency and percentage for relevant subgroups, unless otherwise specified.

**Figure 1:**
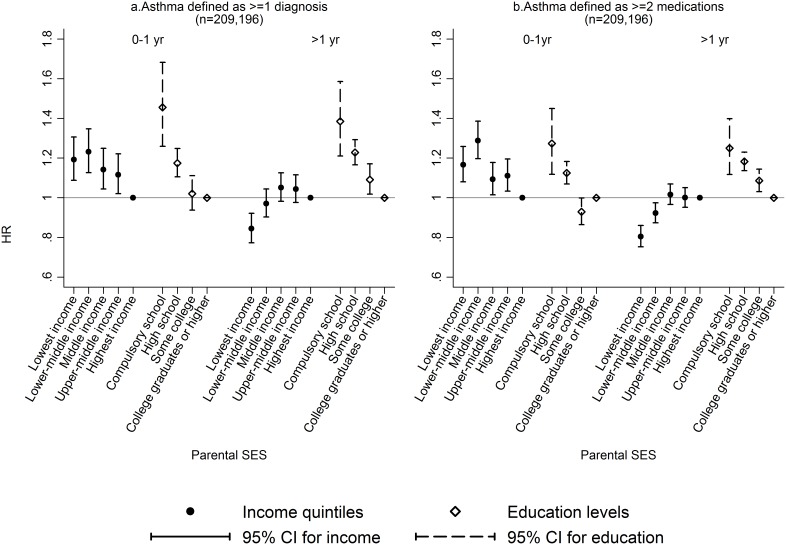
Hazard ratios of childhood-onset asthma by parental SES. Association between parental SES (income and education) and childhood-onset asthma (diagnosis or ≥2 medications) in first year of life and up to 4.5 years.

Figure 1 displays the association between current parental SES and childhood-onset asthma. The proportional hazard assumption did not hold for either asthma definitions (p<0.001). Therefore, we estimated separate effects for the first year of life and the time after the first year for both asthma definitions. During a child’s first year of life, those from the lowest compared to the highest income families were more likely to be diagnosed with asthma (fully adjusted HR 1.19, 95% CI 1.09–1.31) or to dispense ≥2 asthma medications (HR 1.17, 95% CI 1.08–1.26). This association turned negative after the first year of life for lowest income families compared to the highest (fully adjusted HR 0.84, 95% CI 0.77–0.92 for diagnosis, and HR 0.80, 95% CI 0.74–0.86 for medications, both p-values for trend <0.001). Furthermore, children from the lowest compared to the highest educated families were consistently at increased risk of asthma, independent of definition and regardless of age (fully adjusted HR 1.46, 95% CI 1.26–1.68 for children up to 1 year of age and HR 1.39, 95% CI 1.21–1.59 for children aged one or older for asthma diagnosis, all p-values for trend <0.001).

**Figure 2:**
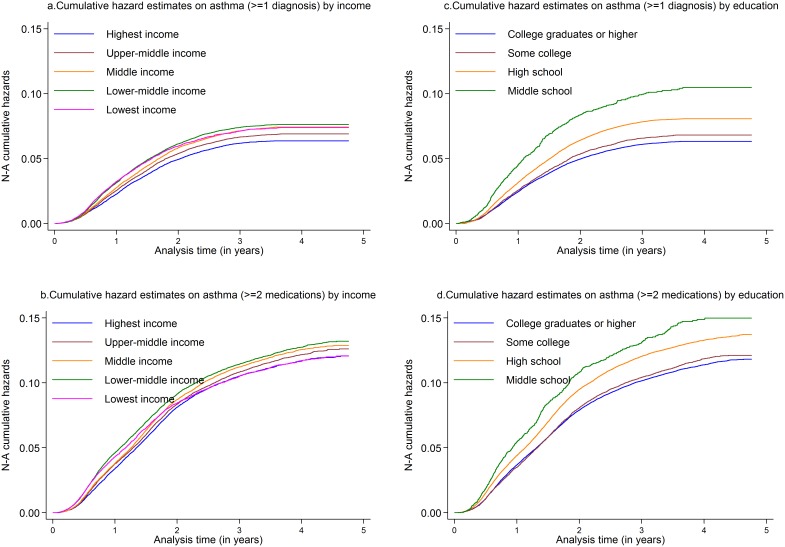
Cumulative hazards of childhood-onset asthma by parental SES. Cumulative hazards of childhood asthma (diagnosis and ≥2 medications) in different SES groups (parental income and education). Estimates adjusted for gender, parity, maternal age, marital status at delivery, healthcare regions, and metropolitan areas.

The cumulative hazards curves for asthma defined as ≥1 diagnosis and as ≥2 prescriptions by income groups (Figure 2 a–b) show that the children in the lowest income group get diagnosis and medications earlier than the others, with a catch up by other income groups around the age of 4 years. Regarding parental education levels (Figure 2 c–d) the initial differences on asthma incidence between SES groups are preserved throughout the follow up. Estimates were similar with adjustment for maternal smoking during pregnancy, low birth weight and preterm birth and there was no effect modification through preterm birth. Risk estimates were also similar for severe asthma defined as ≥3 filled prescriptions within 12 months (data not tabulated). There was no interaction between income and education (lowest p = 0.50).

**Figure 3:**
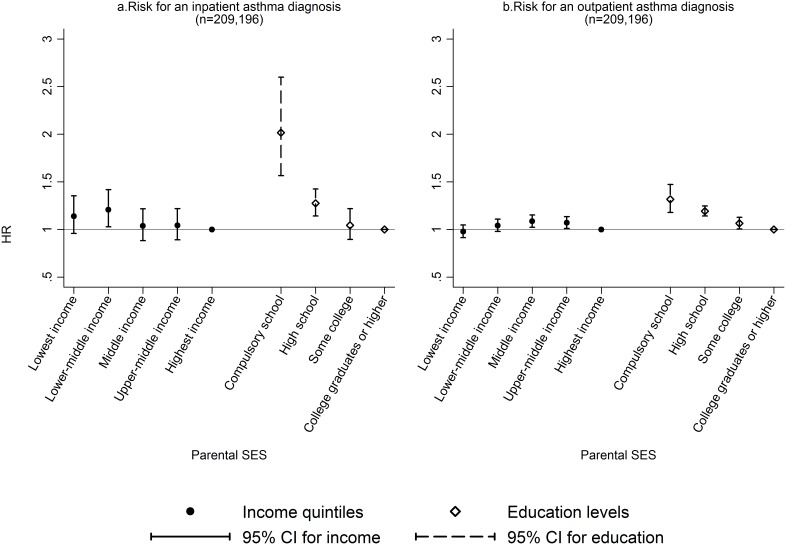
Hazard ratios of inpatient and outpatient asthma diagnosis by parental SES. Hazard ratios and 95% confidence intervals for the association between parental SES (income and education) and inpatient or outpatient asthma diagnoses. The estimates have been adjusted for gender, parity, maternal age, marital status at delivery, healthcare regions, and metropolitan areas.

Figure 3 shows the association between parental SES and risk of receiving an inpatient or outpatient asthma diagnosis. There was no difference in the risk of receiving an inpatient or outpatient diagnosis for children from the lowest income group compared to the highest income group. For parental education, the finding was consistent for both inpatient and outpatient diagnoses, with increased risk of an asthma diagnosis in inpatient care (adjusted HR 2.07, 95% CI 1.61–2.65) as well as outpatient care (adjusted HR 1.32, 95% CI 1.18–1.47) for the lowest education group, compared to the highest education group (both p-values for trend <0.001). There was no effect of parental income or education on risk of a next visit at inpatient care after first diagnosis (results not shown).

**Figure 4:**
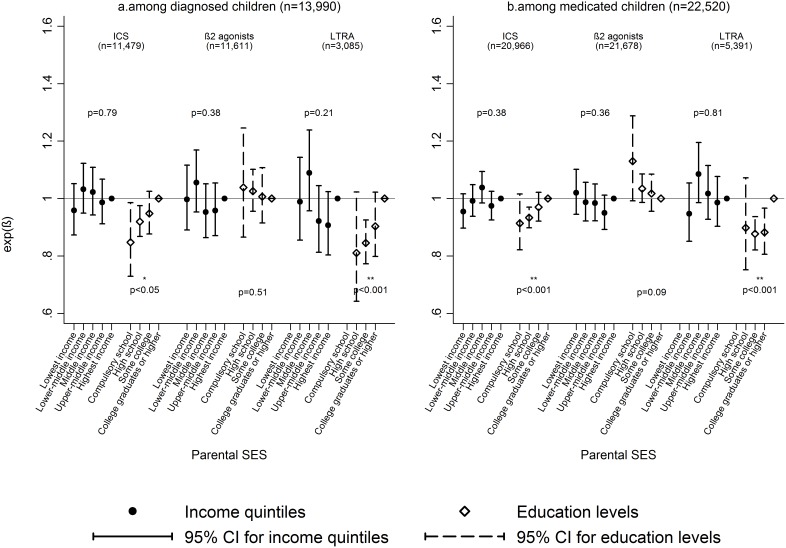
Average doses of asthma medications by parental SES. Average doses (log transformed) of asthma medications (ICS, b-agonists and LTRA) predicted by parental SES (income and education) in the group of children with asthma (diagnosis or ≥2 medications). Estimates in 4a have been adjusted for child age, gender, parity, maternal age, marital status at delivery, healthcare regions, metropolitan areas and those in 4b have been additionally adjusted for asthma diagnosis (yes/no).

Table 2 displays dispensed asthma medications by parental SES among children diagnosed with asthma. Overall, 1,349 out of the 13,990 (9.6%) diagnosed children had not had any dispensed asthma medication since their first asthma diagnosis from NPR, with the highest percentages in the highest income (11.3%) and lowest education groups (9.8%). The percentages of diagnosed patients that had filled prescriptions of ICS and β2-agonists were slightly over 60% and 80%. Around one fifth of diagnosed patients had dispensed medications of LTRA and 2% had combination products. Figure 4 graphically depicts the association between parental SES and average daily doses of different asthma medications among children diagnosed with asthma and children with at least 2 dispensed prescriptions of asthma medications. There was no statistically significant effect of income on average dose of dispensed asthma medication. However, children from less educated families had lower daily doses of dispensed ICS and LTRA, compared to those from the most educated families (college degree or higher). For example, the average (geometric mean) daily dose of ICS was 15% lower (exp(β) = 1.15, p-value for trend = 0.002) in the compulsory school groups compared to children of college graduates, while there was no difference in average daily dose of β2- agonists. Furthermore, among children with at least 2 dispensed medications, the average doses of ICS tended to be lower in families with lower parental education background after adjusting for individual β2- agonist use and diagnostic status (p-value for trend <0.001).

**Table 2:** Frequency of medications consumptions among diagnosed children from different SES groups (parental income and education at diagnosis) from date of first diagnosis of asthma in a Swedish cohort of 211,520 children age 0–4.5.

SES	Number of patients	No. (%) of ICS	No. (%) of β2-agonists	No. (%) of LTRA	No. (%) ofCombinationproducts	No. (%) of nomedications at all
***Income***						
Lowest	2,235	2,004 (64.3)	2,583 (82.9)	661 (21.2)	83 (2.7)	265 (8.5)
Lower-middle	2,980	2,744 (67.7)	2,583 (83.7)	690 (22.4)	86 (2.8)	229 (7.4)
Middle	3,095	1,972 (68.6)	2,430 (84.6)	640 (22.3)	74 (2.6)	247 (8.6)
Upper-middle	2,952	1,781 (69.0)	2,162 (83.7)	591 (22.9)	52 (2.0)	251 (9.7)
Highest	2,720	1,515 (65.0)	1,918 (82.3)	521 (22.3)	46 (2.0)	264 (11.3)
Total	13,990	9,360	11,678	3,103	341	1,349
***Education***						
Compulsoryschool	508	309 (58.1)	436 (82.0)	111 (20.9)	11 (2.1)	52 (9.8)
High school	6,021	3,970 (65.8)	5,050 (83.7)	1,331 (22.0)	156 (2.6)	495 (8.2)
Some college	1,808	1,224 (67.4)	1,504 (83.6)	379 (20.9)	54 (3.0)	175 (9.6)
Collegegraduateor higher	5,640	3,853 (68.8)	4,681 (83.6)	1,280 (22.9)	119 (2.1)	534 (9.5)
Total	13,990	9,360	11,678	3,103	341	1,256

Definition of abbreviations: SES = socioeconomic status; ICS = inhaled corticosteroids; LTRA = leukotriene receptor antagonist.

## Discussion

In this population-based cohort of 211,520 pre-school children, the study results support our hypotheses that there is an association between parental SES and childhood asthma occurrence, level of healthcare use at diagnosis and pattern of medication usage. Interestingly, parental income and education seem to affect asthma outcomes in different ways. Firstly, children from families with lower income or education had higher incidence rates of asthma measured by specialist diagnosis or medication. However, there was a lower incidence in the lowest income group after one year of age, to such an extent that there was a catch-up by the other groups. Secondly, those in families from the lowest education group had an increased risk of in- and outpatient asthma diagnosis and asthma medication. Finally, lower amounts of controller medications were dispensed for children from families with lower parental education.

Although the effect of SES on the development of childhood asthma has been observed in hospital-based and other epidemiological studies [7], [24], [25], asthma diagnosis and medications in relation to age, income and/or education in the general population have not been previously characterized. Reviews conducted by Mielck et al [26] and Rona et al [27] have demonstrated inconsistent findings on the association between SES and childhood asthma overall. However, more recent findings focusing on children at preschool age [8], [9], [28]–[33] showed that those from low SES familial background are more likely to develop asthma, which is partially in line with our result.

Based on our finding on the association between different SES indicators and incident asthma, one possibility is that parental healthcare seeking behaviour, i.e. the income gradient of parents’ request for diagnosis and treatment, differs between the first year of life and later in childhood. Halldórsson et al. found no difference in using primary healthcare for children above 2 years of age, but less specialist care and more inpatient care for those from the lowest income group [34]. Another recent study from Eastern Sweden showed that low-income families received equal child health services from birth until 4 years of age [35]. Thus, the age-varying effect on income in our study remains a challenge because of lacking information on parental choice of healthcare-related cost over time.

Furthermore, as consistent with previous literature [36], [37], our data support the hypothesis that there was an effect of parental education level on asthma diagnosis and medication use among preschool children. However, the question remains why children of more educated parents have less asthma. Admittedly, the mechanism of association has been hypothesized to involve multidimensional factors and only a small part of the education gradient could be attributed to years of parental schooling alone. Despite factors that we controlled for, there are still other potential covariates that are difficult to measure including life style (e.g. breastfeeding) [38] and health literacy [39] in this total population.

Our results also indicated an effect of lower parental income and education on level of healthcare use at asthma diagnosis. This may reflect a tendency for less advantaged groups to seek care at emergency units due to parental stress and need of healthcare services [34], [40], while more advantaged groups may be more prone to have an established relation with a general practitioner or a private outpatient specialist, which is not recorded in the NPR. On the other hand, it is also possible that groups with lower parental education and income are more often admitted to hospital due to their offspring’s symptoms and severity at the first occasion they seek healthcare [41]. Additional analyses, however, did not support any evidence of disparities for a next visit at inpatient care after first diagnosis.

Among children with diagnosed asthma, we found that parental income did not have any influence on average daily doses of all types of medications, which was in contrast to previous findings [16], [42]. This could be due to the equity-promoting medication benefits in Sweden: pharmaceutical expenses for all children under 18 years old are fully reimbursed over a total annual cost of 189 EURO per family [43]. However, there was a worrisome association between parental education and dispenses of controller medications. Less ICS and LTRA were consumed by children in families with lower parental education background, and dispenses of ICS were even lower when adjusting for use of quick-relief medication (for those with confirmed diagnosis and/or at least 2 dispensed medications in whom they should be strongly indicated). Established asthma guidelines [15] and patient education on the importance of adherence to controller medications in young children should be provided and followed up by both clinicians and parents, in order to reduce asthma exacerbation and avoidable healthcare costs. A recent review on adherence to paediatric asthma treatment indicates inconsistent result on non-adherence with familial socioeconomic indicators [44]. However, we did not have access to issued prescription records but only dispensed medications so we were not able to compare the degree of non-adherence through the rate of proper information, prescriptions’ initiating, filling or usage.

The present study is the first to show an association between parental SES and asthma medication purchases for children in a general population. By using register based information on filled prescriptions in pharmacy and diagnosis records from inpatient and outpatient care, we obtained objective measurements on childhood asthma occurrence, healthcare resource usage, and quantities of medication consumption [45]. We also collected register based information on SES and potential confounders, which preclude recall bias.

The current study also has limitations. Firstly, using the NPR for ascertainment of asthma diagnoses has its drawback on differentiating asthma from transient wheezing in preschool children and missing information on children diagnosed in the primary care setting. Instead, we added the alternative definition on dispensed asthma medication as an additional proxy of asthma [22]. However, we cannot rule out the potential misclassification on asthma and bronchitis/bronchiolitis by measuring asthma medications. Secondly, children of parents who were born abroad and migrated to Sweden after 15 years of age were excluded, since their parental education levels may be underestimated systematically from the register. Thus, the results of this study may not apply to these groups of immigrant families in Sweden. Thirdly, parents with illicit working conditions or tax evasion (estimated to 6%) would result in underestimation of income [46], and consequently some of those would be wrongly classified into lower quintiles and we might not capture their actual income.

In conclusion, our population-based study shows that there is an increased risk of early childhood asthma diagnosis among families with lower income and education. Even though it appears that liberal prescription drug benefits in Sweden promote equal use of total medications between lower and higher SES families, the fact that lower SES families dispense fewer of the controller medications and are more often hospitalized at first diagnosis suggests obstacles more subtle than simply cost. The findings call for improved clinical measures to reach all young children in early stage of asthma development. Such approaches might include information and well maintained surveillance of asthma control therapy, asthma severity assessment and improved adherence to international guidelines targeted especially towards groups with lower SES over a long period.
